# Combining Ability in Maize Breeding Programs in Sub-Saharan Africa: A Systematic Review

**DOI:** 10.3390/genes17020168

**Published:** 2026-01-30

**Authors:** Kolawole Peter Oladiran, Pedro Silvestre Chauque, Rogerio Marcos Chiulele, Gift Chinonye Gbaruko, Constantino Francisco Lhamine, Suwilanji Nanyangwe, Mable Kipkoech Chebichii, Mathews Laston Kambani

**Affiliations:** 1 Department of Crop Production, Eduardo Mondlane University, Maputo 1102, Mozambique; chiulele.rogerio@gmail.com (R.M.C.); bycosta.francisco@gmail.com (C.F.L.); suwilanjinanyangwe02@gmail.com (S.N.); mablekipkoech13@gmail.com (M.K.C.); 2 Centre of Excellence in Agri-Food Systems and Nutrition (CE-AFSN), Eduardo Mondlane University, Praca 25 de Junho Edificio da Reitoria 5° andar, Maputo 1102, Mozambique; kambanimathews@gmail.com; 3 Agricultural Research Institute of Mozambique (IIAM), Maputo 1102, Mozambique; 4 Department of Crop and Horticultural Sciences, University of Ibadan, Ibadan 200005, Nigeria; chinonyeebun@gmail.com; 5 International Institute of Tropical Agriculture (IITA), Ibadan 200001, Nigeria; 6 Department of Agricultural Economics, Eduardo Mondlane University, Maputo 1102, Mozambique

**Keywords:** maize, sub-Saharan Africa, combining ability, mating design, hybridization

## Abstract

**Background/Objectives**: Combining ability (CA) analysis is a key tool in maize breeding for developing superior hybrids by evaluating parental genetic potential through general combining ability (GCA) and specific combining ability (SCA). Despite its widespread use, knowledge of how CA techniques help overcome major constraints to maize production in sub-Saharan Africa (SSA) is limited. This review summarizes recent applications of CA analysis in addressing maize breeding challenges across SSA. **Methods**: A systematic literature search was conducted using ScienceDirect, Springer, and Google Scholar for studies published between 2020 and September 2025. Search terms included maize, combining ability, and SSA. The review followed PRISMA guidelines, and 94 studies met the eligibility criteria and were included in the analysis. **Results**: Most studies were conducted in Nigeria (42%), Ethiopia (16%), and Ghana (14%), indicating regional concentration of maize hybridization research within SSA. Yield improvement was the dominant breeding objective across the region. Inbred lines with high GCA were predominantly used as parental materials compared with open-pollinated varieties. The line × tester mating design was the most frequently applied, followed by other mating designs. Across 580 environments, GCA contributed 80%, SCA 19%, and combined GCA/SCA 1% to hybrid performance. The predominance of GCA across traits and environments underscores high additive gene effects, largely due to the high homozygosity of inbred line parents. **Conclusions**: It has been observed in this systematic review that combining ability analysis remains essential for enhancing maize productivity and resilience in SSA by enabling identification of superior parents, efficient mating designs, and development of widely adapted hybrids.

## 1. Introduction

Combining ability (CA) is a cornerstone of crop breeding as it provides insight into the genetic potential of parents and the type of gene action controlling important traits. It refers to the capacity of a parent to transmit desirable traits to its offspring through hybridization, thereby guiding breeders in predicting hybrid performance and designing efficient breeding strategies [1,2,3]. Combining ability is the central base for crop improvement, as it helps identify superior parents for hybrid development [2,4]. This enhances breeders’ ability to select appropriate parents and design effective breeding strategies for producing superior hybrids. The benefits of hybridization (crosses) between two distant parents are gene recombination and heterosis (vigor) of the offspring. Since the genetic value of a parent cannot be reliably predicted from its phenotype alone, their CA is assessed through progeny testing [5,6,7]. Parents that consistently contribute to vigorous, high-yielding hybrids in many crosses exhibit strong general combining ability (GCA), while favorable performance in specific hybrid combinations reflects specific combining ability (SCA) [8,9,10]. GCA is largely associated with additive gene effects, whereas SCA arises from non-additive effects such as dominance and epistasis [8,11,12]. These parameters guide breeders in selecting parents, predicting hybrid performance, and designing efficient strategies [13,14].

The introgression of desirable genes from parents into progenies depends on the nature and number of genes associated with a trait, as well as the relationship between target and secondary traits [15,16,17]. As it has been established, most qualitative (discrete) traits like color, seed coat color, leaf shape, disease, and pest resistance are typically controlled by a few genes (monogenic), whereas quantitative (continuous) traits like plant height, yield, and abiotic stress tolerance are controlled by many genes (polygenic) and they are often linked to other trait loci across the genome [4,15]. This complexity makes the careful selection of parental material and the choice of mating design important for the successful transfer of desired traits during hybridization.

However, the breeder’s ability to achieve genetic gain also depends on how accurately phenotypic values reflect underlying genotypic values. This relationship is quantified through heritability, which measures the proportion of phenotypic variance attributable to genetic factors [18,19,20]. High heritability, particularly when associated with high genetic advance, provides ideal conditions for effective selection and is typically observed in qualitative traits [4,15,21]. Conversely, quantitative traits such as yield are often influenced by additive gene action and environmental interactions [21,22,23]. They tend to show lower heritability across various environmental and management conditions. On the other hand, pest and disease resistance in plants are controlled by a few genes, mostly influenced by dominant gene action or epistatic effects of genes on one another [24,25,26]. Together, combining ability analysis of distant parental lines and heritability estimates in progenies form an essential foundation for identifying promising parents, understanding trait inheritance, and accelerating genetic improvement, particularly in maize.

In maize improvement, mating designs such as line × tester, diallel (half or full), top-cross, and the North Carolina designs (I, II, III) are commonly employed to partition GCA and SCA effects and to reveal the underlying gene action of important target traits for breeding objectives [27,28,29]. These designs remain particularly relevant in sub-Saharan Africa (SSA), where maize (*Zea mays* L.) is the most widely cultivated cereal crop. More than half of the agricultural land in SSA is devoted to maize, underscoring its importance for food security and rural livelihoods [30,31,32]. Maize provides over 30% of the daily calorie intake, ranging from 30 to 50 g per person per day [33,34]. In sub-Saharan Africa, millions of households rely on maize not only for food and nutritional security but also as a key source of income to meet their essential needs [31,35]. In many rural communities, maize serves as a major protein source despite its deficiency in lysine and tryptophan [36,37,38,39]. However, productivity is constrained by limited access to improved varieties, biotic and abiotic stresses, and highly variable agroecological conditions [40,41,42]. Combining ability studies offer crucial insights for addressing these challenges by enabling breeders to evaluate genetic potential, identify promising parental lines, and understand trait inheritance [43,44,45]. Effective use of GCA and SCA effects can accelerate the development of high-yielding, stress-tolerant, disease-resistant, and nutritionally enhanced hybrids adapted to SSA production systems.

Despite growing research attention on SSA, knowledge gaps remain regarding the predominant mating designs employed, the traits most frequently targeted, and the relative importance of GCA and SCA effects on the focused traits. Therefore, the objectives of this review are (i) to analyze the use of CA studies for targeted traits with emphasis on grain yield, abiotic stress tolerance, and biotic stress (e.g., disease resistance), (ii) to identify the most commonly used mating designs in CA studies, and (iii) to evaluate the relative contributions of GCA and SCA across studies. By consolidating the available evidence, this review provides breeders and other researchers with insights into methodological trends and trait focus, supporting the design of more efficient maize breeding strategies in SSA.

## 2. Materials and Methods

### 2.1. Scope of the Study

The scope of this systematic review is limited to studies on the combining ability of maize in sub-Saharan Africa (SSA). The review protocol followed the PICOTS framework. The review focused on hybrid maize (*Zea mays* L.) populations developed through controlled hybridization of parental materials and evaluated across sub-Saharan Africa (SSA). Intervention involved the application of established mating designs, such as the line × tester, diallel, North Carolina, and top-cross schemes, to estimate combining ability effects. Comparators included variations in study objectives, mating designs, and parental materials used to generate hybrids. The outcomes assessed were the estimates of general combining ability (GCA) and specific combining ability (SCA), as well as their relative contributions to hybrid performance. The review covered research articles published between January 2020 and December 2025, thereby allowing the inclusion of studies conducted in earlier years (as far back as 2013) but published within the defined timeframe. This approach enabled a broader synthesis of research on maize hybridization in sub-Saharan Africa (SSA). Evaluations were conducted across multiple seasons, and details of all trials are reported in the Supplementary Materials. The study settings comprised experimental trials conducted under field or controlled environments (such as screenhouses), encompassing different seasons, water management regimes, and a range of abiotic and biotic stress conditions.

### 2.2. Review Protocol and Reporting Standards

This systematic review was undertaken in line with the PRISMA guidelines and followed a protocol registered in the Open Science Framework (OSF; https://doi.org/10.17605/OSF.IO/K7F9C). The registration was performed to enhance transparency in the processes of study selection, data extraction, and evidence synthesis.

### 2.3. Search Strategy

This systematic review adopted a structured approach to identify, screen, and analyze studies on combining ability in maize across SSA. A comprehensive search was conducted in three major databases: Springer, ScienceDirect, and Google Scholar (using publishorperish version 8.17) to access peer-reviewed journal articles as well as master’s and doctoral dissertations. The search strategy relied on structured strings with Boolean operators, specifically, (“Combining ability”) AND (“maize” OR “*Zea mays*”) AND (“Africa”) AND (“2020” OR later). The Boolean operators were developed to capture a wider range of studies across SSA specifically using “Africa” as a key word, since “SSA” generated a smaller number of relevant studies during the first search across the accessible databases. The search for eligible studies was completed in September 2025. The use of Google Scholar enabled the inclusion of a broad range of studies, including theses and dissertations from university repositories as well as articles from multiple journal platforms, as documented in the study data extraction sheet and reviewed articles bibliography provided in the Supplementary Materials (https://doi.org/10.17605/OSF.IO/K7F9C). Retrieved records were identified and verified using their Digital Object Identifiers (DOIs), URL links attached to each included study. This also catered for articles from multiple journal websites including MDPI, Wiley, BMC, Frontiers, Plos One, etc.

### 2.4. Eligibility Criteria

In order to ensure a comprehensive and focused synthesis of the combining ability studies on maize across sub-Saharan Africa, the following criteria were developed and applied.

#### 2.4.1. Inclusion Criteria

The inclusion criteria for this systematic review included studies that involve maize (*Zea mays* L.) hybridization (crossing) with a report of a recognized mating design for hybrid development, including line × tester, diallel, North Carolina, or top-cross designs. Eligible studies were required to report and clearly specify combining ability effects, particularly general combining ability (GCA) and specific combining ability (SCA), of parental materials in relation to hybrid performance. Only peer-reviewed journal articles, dissertations, theses, or conference proceedings providing sufficient methodological detail were considered. In addition, studies had to be conducted within sub-Saharan Africa (SSA) and published between 2020 and 2025. Only studies reported in English were considered, as translation was not possible for studies in other languages.

#### 2.4.2. Exclusion Criteria

The following exclusion criteria were applied in this systematic review to ensure relevance and methodological consistency. Studies were excluded if they did not involve the evaluation of hybrid maize or lacked a clearly defined mating design or crossing scheme. Publications without primary data, such as review articles, opinion pieces, editorials, or interviews, were also excluded. In addition, studies that assessed hybrids using methods other than combining ability estimation, or that did not report general or specific combining ability effects, were not considered for inclusion.

### 2.5. Selection Process

The study selection process was systematically structured to ensure the inclusion of only relevant and high-quality research aligned with the objectives of this review. Literature searches were conducted using Google Scholar, ScienceDirect, and Springer, after which all retrieved records were imported into Microsoft Excel for organization, preliminary sorting, and removal of duplicates. The individual records were assessed by two reviewers independently, while validation was performed by a third reviewer and a review expert mentioned in the Acknowledgments. Titles, abstracts, and crop species were used as initial screening criteria to exclude studies that were clearly outside the scope of hybrid maize research. Articles that met the basic inclusion requirements were subsequently subjected to full-text screening to confirm eligibility, with emphasis on the use of clearly defined mating designs, such as line × tester, diallel, North Carolina, or top-cross, and the reporting of combining ability estimates in terms of general combining ability (GCA), specific combining ability (SCA), or combined GCA/SCA effects. Reference management and screening were supported using Mendeley and Zotero to catalog publications, review abstracts and full texts, and organize documents for data extraction, thereby enhancing the consistency, transparency, and reproducibility of the study selection process.

### 2.6. Data Collection Process

For eligible study, detailed data were extracted on several aspects of the research. This was performed independently by 3 reviewers to prevent bias and minimize mistakes. Discrepancies were discussed among the reviewers where necessary, and conclusions were made. Inspection of collected data was performed by an external researcher named in the Acknowledgments. Data were collected on the objective of the study, study traits, the mating design applied, the number and type of parental materials, and the progenies evaluated. Additional information collected covered experimental design, number of replications, country of study, geographic coordinates, site description, year and season of experimentation, and prevailing environmental and field conditions (classified as natural/rainfed, optimum/non-stressed/well-watered, and stressed/infested). Crucially, the type of combining ability reported (GCA, SCA, or both) was also recorded to facilitate comparative analysis. The final dataset was carefully reviewed and checked for consistency by all reviewers. This system of data collection ensured that eligible studies met the inclusion criteria and the objective of this systematic review.

### 2.7. Data Items

The study reviewed focused on the use of a combining ability approach in addressing major challenges facing maize improvement and production across SSA. To achieve this, data on study distribution across SSA region, objective of the studies (trait of focus), mating designs, parent material, hybrid evaluation (numbers and types of environment/location/sites), experimental design, and combining ability estimates in terms of GCA and SCA were synthesized. All eligible studies reported each of the data items for effective estimation of combining ability and development of improved hybrids across SSA.

#### 2.7.1. Missing Data

Due to differences in research objectives and evaluation conditions used in estimating combining ability across the included studies, a high level of heterogeneity was observed in the type and number of traits measured. In addition, the number of parental materials, as well as the sites and seasons of evaluation, varied considerably among the selected studies. In most cases, combining ability estimates were aggregated by the respective study authors and reported in terms of the predominant gene effects in their discussions and conclusions. Furthermore, using the general formula for combining ability estimates (Baker’s ratio) and estimates of narrow-sense heritability where high values indicate additive gene action, the type of gene action was identified. Consequently, combining ability estimates were extracted and reported in a generalized manner, represented as GCA, SCA, or GCA/SCA, to allow aggregation across studies in accordance with each study author’s interpretation.

#### 2.7.2. Geographic Limitations

This review considered the sub-Saharan Africa region which includes West, Central, East, and Southern Africa, where maize is the most widely cultivated cereal crop. These subregions host the majority of international research institutes actively involved in maize breeding and improvement programs as well as seed companies. Mapping study coordinates allowed the review to assess not only the geographical distribution of combining ability research but also the extent to which progress in breeding programs correlates with maize yield outcomes across the region over the review period.

### 2.8. Risk of Bias for the Included Studies

The selected studies were assessed for risk of bias using robvis, a visualization tool developed for use in systematic reviews. Using the ROBIN-I tool for risk-of-bias assessment, a total of 94 studies were evaluated across seven bias domains, as described by [46]. These domains included (1) bias due to confounding factors, (2) bias due to selection of participants, (3) bias in classification of interventions, (4) bias due to deviations from intended interventions, (5) bias due to missing data, (6) bias in measurement of outcomes, and (7) bias in selection of the reported results. Each study was independently assessed by three reviewers and classified under each domain as having low, moderate, serious, or critical risk of bias, following the criteria outlined by [46]. The structured assessment across the seven domains enabled a systematic evaluation of study quality and suitability for inclusion in the review [46,47]. Potential sources of systematic differences were minimized by carefully considering environment (field versus screenhouse trials) as a confounding factor, parental line identity and source as participant selection, mating design as the intervention, evaluation conditions (optimal or stress) as deviations from intended interventions, completeness of recorded data as missing data, reporting of variance components as outcome measurement, and completeness of combining ability estimates as selective reporting [46]. Discrepancies among reviewers were discussed and resolved through consensus, ensuring a transparent, unbiased, and rigorous approach to risk-of-bias assessment in this systematic review.

### 2.9. Effect Measures

For this systematic review, outcomes were reported as a summarized measure for each study rather than as generalized quantitative estimates. This approach was adopted because of the wide variation among studies in research objectives, number and type of traits evaluated, and number of locations and seasons used for trial evaluation, which resulted in substantial heterogeneity across studies. Consequently, individual studies aggregated their results across traits and environments and reported the predominant combining ability (CA) effects, expressed as general combining ability (GCA), specific combining ability (SCA), or a combined GCA/SCA effect. This method of outcome reporting was therefore used to ensure consistency and comparability in synthesizing evidence across the included studies.

### 2.10. Synthesis Methods

The extracted data were synthesized using descriptive analysis because the high level of heterogeneity among the included studies prevented the use of pooled effect or meta-analytic methods. This heterogeneity arose from differences in study objectives, outcome measures, traits evaluated, and experimental conditions. As a result, findings were summarized and presented using charts and tables to illustrate variations across the synthesized data categories. Meta-analysis was not feasible due to inconsistent reporting of outcome measures and evaluation conditions across studies. Instead, results were synthesized using a structured narrative approach in accordance with PRISMA guidelines for heterogeneous datasets, ensuring a clear linkage between individual studies and their contributions to the overall synthesis. Geographic mapping of the studies was performed using QGIS Desktop version 3.38.1, while plots and tables illustrating data distribution and relationships were generated using Microsoft Excel. Reports are presented on (i) geographical distribution of studies, (ii) study objectives, (ii) mating designs, (iv) parent materials, and (v) CA estimations

## 3. Results

### 3.1. Study Selection

**Figure 1:**
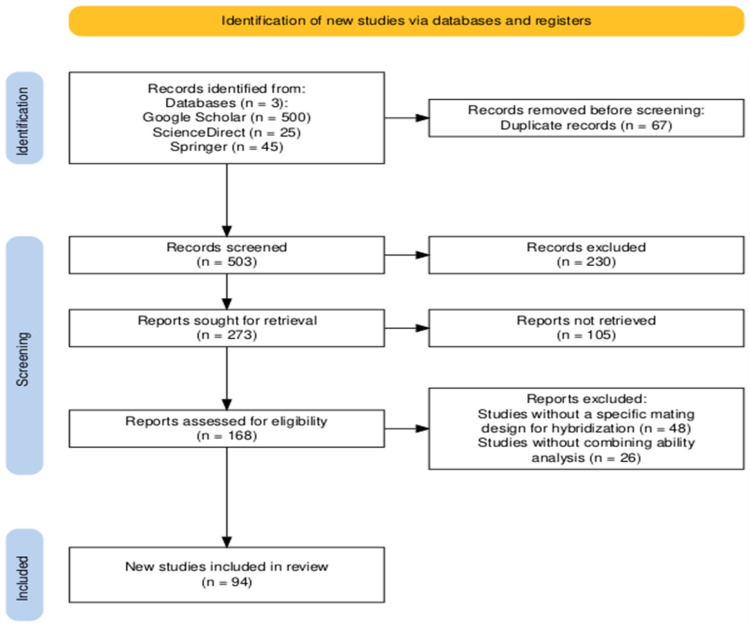
PRISMA flow diagram of the selected studies; the new studies are the those that met the eligibility criteria according to the PICOTS framework.

The literature search identified a total of 570 records from three electronic sources: Google Scholar (*n* = 500), ScienceDirect (*n* = 25), and Springer (*n* = 45). Duplicate records (*n* = 67) were removed using Microsoft Excel (2021). The resulting 503 unique records were cataloged using Mendeley Desktop (version 2.141.2) and Zotero (version 7.0.30) for title, crop species, and abstract screening. During this screening stage, 230 records were excluded for failing to meet the PICOT criteria. Consequently, 273 reports were sought for retrieval, of which 168 full-text articles were successfully retrieved and assessed for eligibility. Following full-text screening, 48 studies were excluded due to the absence of a clearly defined mating design for hybridization, while 26 studies were excluded because they did not report combining ability analysis. Ultimately, 94 studies met the eligibility criteria and were included in the final synthesis. The study selection process is illustrated in the PRISMA flow diagram (Figure 1).

### 3.2. Excluded Studies

During the eligibility assessment for synthesis, several studies initially met the selection criteria but were subsequently excluded because of the way outcomes were measured and reported. In particular, some studies relied primarily on molecular analyses for hybrid classification, which did not explain the average performance of the parental lines in relation to hybrid performance under evaluation. Although these studies employed recognized mating designs and clearly described parental material selection, they did not report parental contributions in terms of general combining ability (GCA) and specific combining ability (SCA). For example, in study [48], 72 hybrids derived from crosses between 36 lines and two testers. The inbred lines were classified into four groups without reporting the average parental performance in hybrid combinations. Similarly, study [49] used DArTseq SNP markers to classify 90 hybrids generated from crosses between 30 inbred lines and three testers into two clusters, but did not report the influence of parental lines on hybrid performance across clusters. Such studies, and others with similar limitations, were therefore excluded.

In addition, some studies evaluated hybrids solely based on heterosis relative to standard checks, with emphasis on identifying high-yielding varieties rather than partitioning parental genetic effects. In these cases, parental contributions to hybrid performance were not quantified using combining ability parameters. This was observed in studies [50,51,52,53,54], where average yield performance was compared only among hybrids and checks. Consequently, studies within this category were excluded from the final synthesis.

### 3.3. Study Characteristics

The included studies varied widely in both number and research objectives across countries within the sub-Saharan Africa (SSA) region. A total of 94 studies were conducted in 13 countries, with considerable variation in the number of studies per country as well as in the number of hybrid evaluation trials reported. Eight distinct research objectives were identified, of which four were addressed independently, while the remaining objectives were examined in combination with one or two additional objectives. Three types of mating designs were reported across the studies, and parental materials were broadly classified into two groups. Hybrid evaluations were conducted under both optimum and stress environments, as detailed in the Section 3.

### 3.4. Risk-of-Bias Assessment

Risk of bias was assessed using a structured, seven-domain-based approach adapted for combining ability and hybrid evaluation studies [46]. Each study was independently evaluated across seven bias domains. To reduce subjectivity, explicit qualitative and semi-quantitative criteria were defined for assigning studies to one of four bias levels: *low*, *moderate*, *serious*, or *critical*. Studies rated as *critical* in any domain were excluded during screening [46].

#### Bias Domains and Assessment Criteria

**Bias due to confounding**

This domain evaluated the adequacy of environmental control and experimental efficiency, including soil characterization, water management, cultural practices, experimental design, and replication.

- **Low risk**: Three or more replications, clearly described experimental design, and evaluation across two or more environments or seasons.
- **Moderate risk**: Two replications and/or single-environment (screenhouse) trials with adequate environmental description/control.
- **Serious risk**: Single-location trials with unclear management practices or insufficient environmental characterization.
- **Critical risk**: No replication or absence of basic experimental design information.

**Bias due to selection of participants (parent materials)**

Assessment was based on the transparency and completeness of parental material description.

- **Low risk**: Full description of parental origin, breeding history, and selection procedure.
- **Moderate risk**: Partial description (e.g., source identified but breeding history unclear).
- **Serious risk**: Parental materials derived from pre-existing populations without methodological detail.
- **Critical risk**: No description of parental sources or selection procedures.

**Bias in classification of interventions (mating design and trial evaluation)**

This domain assessed the clarity, appropriateness, and consistency of the mating design and trial evaluation as experimental interventions to evaluate hybrid performance and combining ability.

- **Low risk**: The mating design and evaluation environment(s) were clearly defined, consistently applied, and well aligned with the study objectives.
- **Moderate risk**: The mating design and/or environment were described but lacked full justification or consistency across trials or locations.
- **Serious risk**: The mating design and/or environmental conditions were inconsistently implemented, limiting interpretability of combining ability estimates.
- **Critical risk**: The mating design and/or evaluation environment were not described preventing reliable reporting.

**Bias due to deviations from intended interventions**

Studies were evaluated for consistency between stated objectives and implemented methodology in terms of testing hybrid performance.

- **Low risk**: Methods fully aligned with objectives.
- **Moderate risk**: Minor deviations not affecting interpretation.
- **Serious risk**: Major inconsistencies between objectives and methods.
- **Critical risk**: Fundamental methodological flaws.

**Bias due to missing data**

Assessment focused on the availability of core analytical information: objectives, parental materials, mating design, GCA/SCA contributions, and other variance components.

- **Low risk**: Complete reporting of all required components.
- **Moderate risk**: One component missing but inferable.
- **Serious risk**: Two or more key components missing.
- **Critical risk**: Absence of GCA/SCA results or mating design.

**Bias in measurement of outcomes**

This domain assessed the clarity and completeness of combining ability estimates.

- **Low risk**: Explicit reporting of GCA and SCA estimates, variance components, and derived parameters (e.g., Baker’s ratio, narrow-sense heritability).
- **Moderate risk**: GCA and SCA reported without full variance decomposition.
- **Serious risk**: Only qualitative interpretation of combining ability.
- **Critical risk**: No quantitative CA estimates.

**Bias in selection of reported results**

Evaluation was based on the consistency between reported results and study conclusions.

- **Low risk**: Conclusions directly supported by quantitative CA results.
- **Moderate risk**: Limited supporting data.
- **Serious risk**: Selective reporting favoring specific outcomes.
- **Critical risk**: Conclusions unsupported by presented data.

### 3.5. Study Distribution

**Figure 2:**
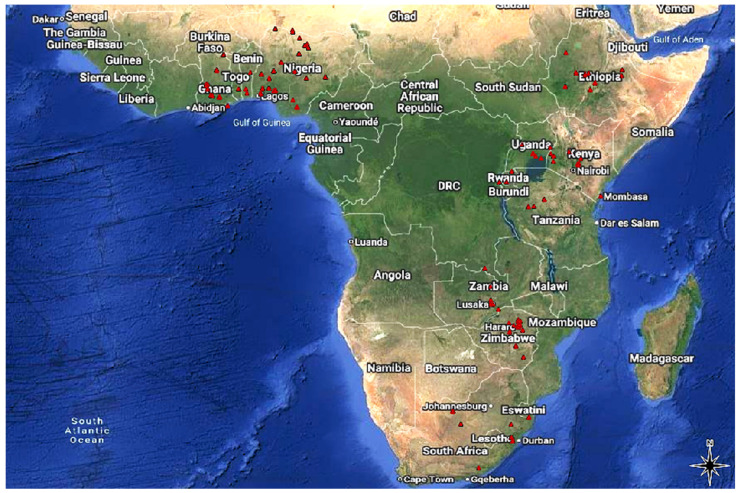
Study (location) distribution map across Africa (the red dots denote the trial location within the country).

**Figure 3:**
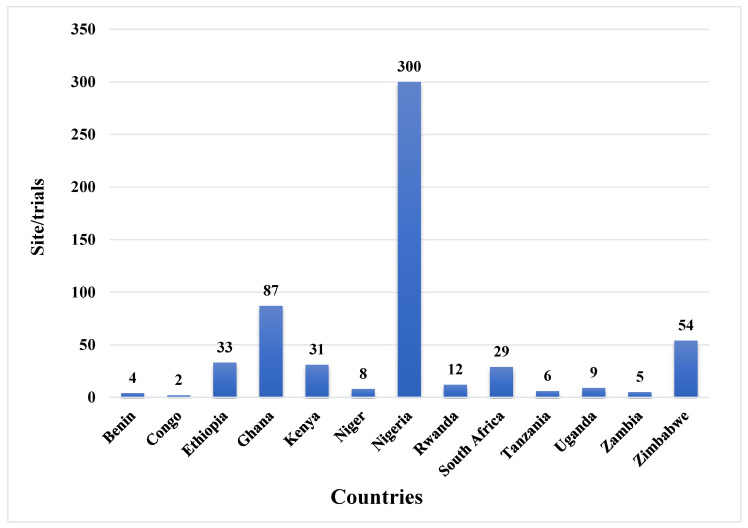
Study evaluation sites across different countries; hybrid evaluation was conducted for different objectives under varying environmental conditions for their performance across countries.

**Figure 4:**
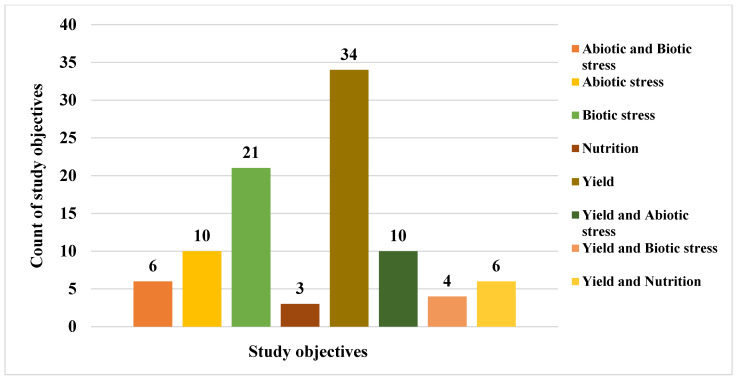
Research objectives of the reviewed studies.

The distribution of the selected study locations reflects the broader pattern of maize research across sub-Saharan Africa (SSA) (Figure 2). The 94 eligible studies were conducted across 13 countries, with varying distribution among them. Nigeria (39 studies), Ethiopia (15), and Ghana (13) recorded the highest number of studies (Table 1). These studies were implemented under diverse management practices and research objectives across multiple environments like location/site, prevailing seasons, and field conditions (optimum and stressed) to evaluate hybrid performance. In total, 580 hybrid evaluation trials were conducted across the 13 SSA countries, with Nigeria accounting for the highest number (300 trials), followed by Ghana (87) and Zimbabwe (54). In contrast, Congo (2 trials), Benin (4), and Zambia (5) recorded relatively few trials, as illustrated in Figure 3 and Figure 4.

### 3.6. Objectives of Selected Studies

**Figure 5:**
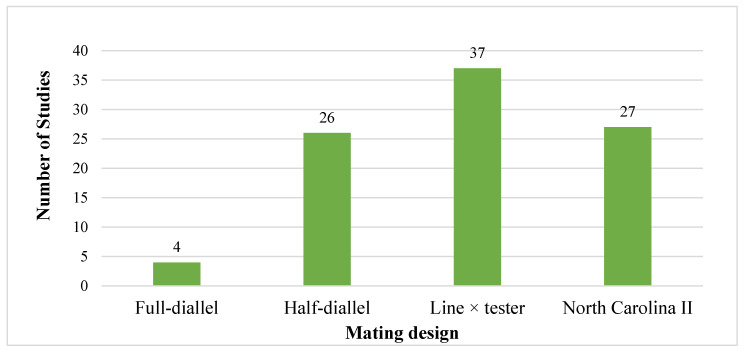
Mating designs used for hybridization across the reviewed studies.

The selected studies addressed a wide range of challenges affecting maize production across SSA. These challenges include abiotic factors such as drought, heat, and low soil nitrogen; biotic factors such as pests and diseases; nutrient deficiencies; and generally low yield. To mitigate these constraints, the reviewed studies demonstrated the extent of research efforts and commitment applied across different locations in SSA. From the analysis, nine major categories of research objectives were identified, with some studies combining two related objectives. These include abiotic stress, abiotic and biotic stress, biotic stress, nutrition, yield, yield and abiotic stress, yield and biotic stress, and yield and nutrition. The distribution of these objectives is shown in Figure 5.

Out of the 94 eligible studies, 54 studies evaluated hybrid maize varieties with the primary objective of improving yield in combination with other goals (Figure 5). This finding underscores that, despite progress in maize improvement programs across SSA, yield enhancement remains the central breeding priority. Abiotic stresses such as drought, heat, and low soil nitrogen, along with biotic factors including pest and disease pressure, continue to limit the yield potential of many released maize varieties. The results also revealed that research targeting biotic stress tolerance is on the rise. This is largely due to the emergence and spread of more aggressive insect pests and virulent pathogens across SSA. Notable examples include fall armyworm (*Spodoptera frugiperda*), *Striga* spp., maize streak virus, and maize lethal necrosis, all of which remain serious threats to maize production.

### 3.7. Mating Design and Parental Materials

**Figure 6:**
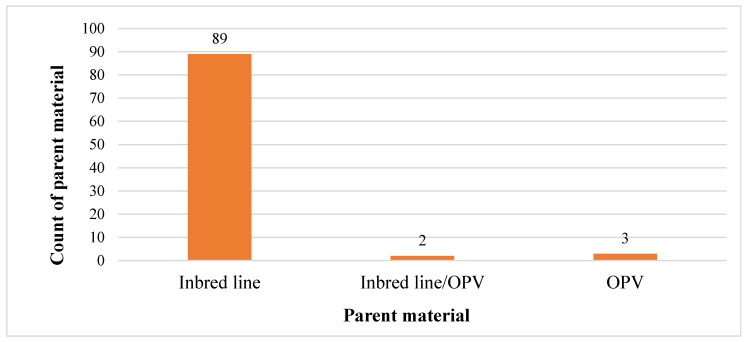
Parent material used for hybridization across the reviewed studies (OPV: open-pollinated variety).

**Figure 7:**
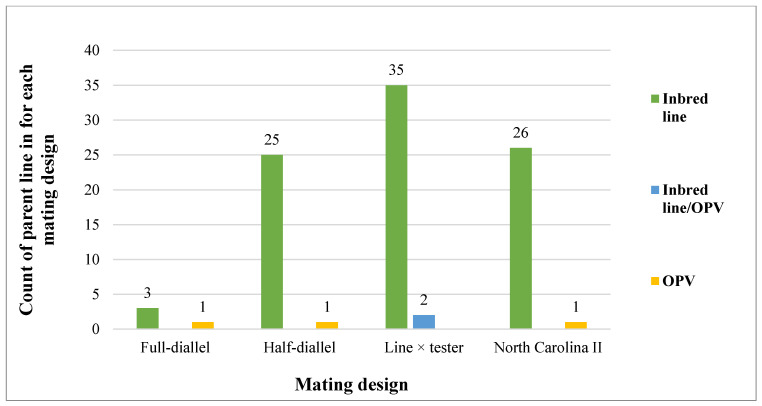
Interaction between mating design and parent materials across the reviewed studies (OPV: open-pollinated variety).

**Figure 8:**
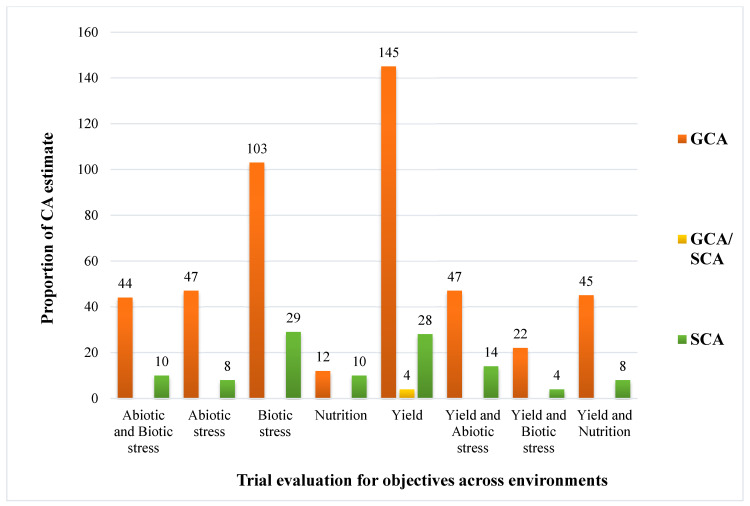
Combining ability contributions for objectives across the environments (GCA, general combining ability; SCA, specific combining ability).

Figure 6, Figure 7 and Figure 8 illustrate the types of mating designs, parental materials employed, and their relationship in the selected studies. Four mating designs were identified: full-diallel (4), half-diallel (26), line × tester (37), and North Carolina II (27). The parental materials consisted predominantly of inbred lines and only in 5% of the studies included open-pollinated varieties (OPVs). Across the reviewed studies, both parental materials (inbred line and OPVs) were pre-screened for all the target traits before hybridization (crossing). In total, 89 studies (about 95%) utilized inbred × inbred crosses, 3 (3%) studies involved OPV × OPV crosses, and 2 (2%) studies combined inbred lines and OPVs as parents.

The line × tester mating design accounted for the highest proportion among the designs used, featuring 37 studies in total (35 involving inbred line crosses and 2 involving inbred line × OPV crosses). Notably, it was the only design employed to generate maize hybrids through crossing between inbred lines and OPV materials. The full-diallel design was reported in 4 studies (3 involving inbred lines crosses and 1 involving OPV parents), while the half-diallel design was used in 26 studies (25 involving inbred lines crosses and 1 involving OPV parents). In these designs, inbred lines and OPVs were used separately in the hybridization process, and there were no intercrosses between the two parental groups. Additionally, the North Carolina II (NCII) design was reported in 27 studies (26 involving inbred lines crosses and 1 involving OPV parents). Similarly to the diallel designs, no intercrossing between inbred lines and OPVs was reported in studies involving the NCII mating scheme.

### 3.8. Hybrid Trial Evaluation

Based on the objectives and target traits of the studies analyzed, the developed hybrid materials were evaluated under different environmental conditions to assess their performance in relation to the specific stresses they were developed to address. A total of 580 trial evaluations were conducted in an open field, while 6 trials were conducted in a screenhouse. Across all reviewed studies, the experimental designs adopted included the alpha lattice design (77 studies) and the randomized complete block design (RCBD) (17 studies). All experiments were replicated two to four times across sites and seasons to ensure data reliability.

Hybrid progenies were tested under various stress conditions, including drought, heat, low soil nitrogen, acidic soils, artificial and natural disease inoculation, pest infestation, and combined stresses. Stress conditions were introduced through different and specific field management practices. Under Striga infestation (artificial or natural), nitrogen fertilizer (optimum) application was delayed to encourage Striga germination and competition with maize. Low soil nitrogen conditions were created through continuous cropping and complete removal of crop residues to deplete soil nitrogen. This was followed by sub-optimal (low) nitrogen application to assess nitrogen-use efficiency of hybrid maize. For disease and pest screening, including fall armyworm (FAW), maize streak virus (MSV), maize lethal necrosis (MLN), and turcicum, trials were located in areas with high natural incidence or by introducing vectors either in confinement (screenhouse) or in an open field. Drought was induced by withdrawing irrigation during critical growth stages, and most trials were conducted during the dry season, when rainfall is minimal. Optimum conditions were ensured through optimum rainfed conditions and irrigation. Heat stress was achieved by establishing the trials in the region with a high temperature range during the cropping season. Most of the research was conducted in an open field with minimal environmental effects, while some were conducted in a screenhouse to prevent environmental influence on the results. These strategies ensured effective identification of genotypes with enhanced tolerance or resistance to the specific stress. A summary of the environmental conditions, categorized as pest and disease stress, water/heat stress management, nitrogen fertilizer, and type of experiment (location), are presented in Table 2.

### 3.9. Combining Ability (CA) Analysis

The reviewed studies addressed diverse breeding objectives, which varied widely in both numbers and types of traits evaluated. The results of the CA analysis for traits measured in individual studies were aggregated by the authors and the relative proportions of GCA and SCA are reported in the results, discussion, and conclusion sections. Consequently, for this review study, reports of combining ability estimates in terms of GCA and SCA were synthesized descriptively and reported as either GCA, SCA, or combined GCA/SCA effects across studies and environment. This is based on the interpretations, discussions, and conclusions reported by the respective authors. These synthesized outcomes are summarized in the data extraction sheets provided in the online database (https://doi.org/10.17605/OSF.IO/K7F9C).

Table 3 summarizes the interaction between parental materials and combining ability estimates across all hybrid evaluation trials. Studies involving inbred lines alone were evaluated in 549 trials, of which 452 reported significant GCA effects, 93 reported SCA effects, and 4 reported combined GCA/SCA effects. Trials involving crosses between inbred lines and open-pollinated varieties (OPVs) were conducted across 18 environments, with 7 studies reporting GCA effects and 11 reporting SCA effects, and no reports of combined effects. Trials involving OPV parental materials alone were evaluated in 13 trials, with 6 reporting GCA effects and 7 reporting SCA effects. These results include studies in which only GCA, only SCA, or combined GCA/SCA effects were observed consistently across all environments, as well as studies in which both GCA and SCA effects were detected across different environments. This result explains the different responses of similar genotypes under different environmental conditions, which is referred to as genotype–environment interaction (GEI). For example, one study [55] reported GCA effect under non-Striga infestation and SCA effect under Striga infestation. These patterns are further interpreted in relation to study objectives and evaluation environments.

Across all the reviewed studies, breeding objectives, and 580 evaluation trials, GCA contributed more to the total variation observed than the SCA and the combined GCA/SCA effects reported. This indicates that additive gene effects were predominant in the inheritance of most traits. The highest contribution of GCA (145) was recorded for yield, followed by biotic stress (103) and abiotic stress as well as the combination of yield and abiotic stress (47). The highest SCA contribution was observed for biotic stress (29), followed by yield (28) and yield and abiotic stress (14).

**Figure 9:**
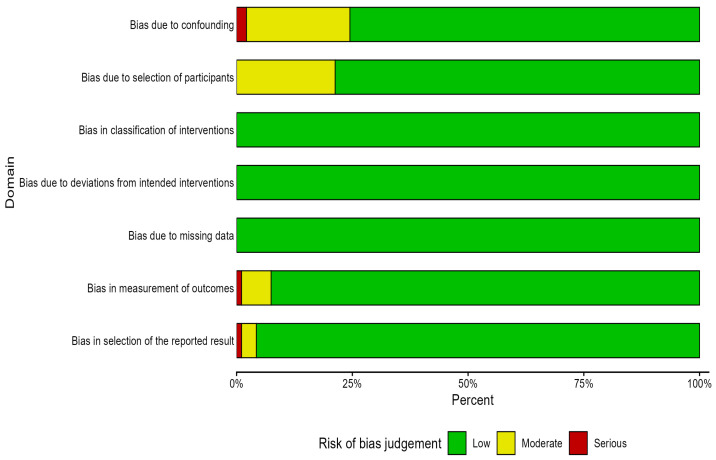
Overall risk-of-bias assessment of the 94 studies. The individual risk-of-bias assessment is provided as a Supplementary Materials.

The combined GCA/SCA effects were observed only for yield, representing equal contributions of both additive and non-additive gene actions to the hybrid performance. This pattern suggests that hybrid performance was largely influenced by the average additive effects of genes contributed by both parents, rather than by non-additive interactions such as dominance or epistasis. Figure 9 below illustrates the contribution of different combining ability effects across the reported breeding objectives.

## 4. Results of Syntheses

### 4.1. Risk of Bias in Studies

The 94 included studies were evaluated for risk of bias using the ROBINS-I framework, with emphasis on combining ability estimates for parental selection. Quality assessment was structured across seven domains: (i) bias due to confounding, which considered evaluation conditions such as experimental design, replication, soil properties, and moisture levels; (ii) bias in selection of participants, assessing the type, traits, number, and source of parental lines; (iii) bias in classification of interventions, evaluating the clarity and appropriateness of mating designs, including line × tester, diallel, and North Carolina; (iv) bias due to deviations from intended interventions, examining whether evaluation environments allowed proper expression of target traits such as drought tolerance, nitrogen-use efficiency, and pest or disease resistance; (v) bias due to missing data, focusing on the completeness of reported variance components used in combining ability estimation; (vi) bias in measurement of outcomes, assessing the reporting of combining ability effects in terms of GCA, SCA, or combined GCA/SCA and narrow-sense heritability; and (vii) bias in selection of the reported results, evaluating consistency between reported results and the authors’ conclusions. Assessments were independently carried out by two reviewers, with discrepancies resolved through consensus. Some risk of bias due to confounding was observed, mainly due to limited environmental descriptions, but such studies were retained where the effect was considered minor. Studies lacking clear information on parental material sources or well-defined mating designs were excluded, while those with complete pedigree information were rated as low risk and those with partial but well-described sources as moderate risk. The overall risk-of-bias assessment is summarized in the traffic-light plot presented in the Supplementary Materials.

### 4.2. Synthesis of Study Distribution Result

The selection of eligible studies in this systematic review is centered on the objective of understanding the trend of research on combining ability for maize improvement across SSA. The distribution of studies reflects the level of participation and involvement of different countries in maize improvement research across sub-Saharan Africa (SSA). The economic significance of maize and its vital contribution to food security in the region underscore the need for sustained research efforts aimed at enhancing the livelihoods of stakeholders engaged in its production [32,42]. The highest numbers of studies were conducted in Nigeria, Ethiopia, and Ghana, indicating a strong research presence and institutional capacity in these countries. This pattern also mirrors the location and influence of major research organizations such as the International Institute of Tropical Agriculture (IITA), the International Maize and Wheat Improvement Center (CIMMYT), the West Africa Centre for Crop Improvement (WACCI), and prominent seed companies such as SeedCo [56]. They all play significant roles in maize improvement programs.

However, the observed variation in the number of studies among countries does not always correspond with national maize production levels. For instance, Kenya, South Africa, and Tanzania, despite being among the top maize-producing countries in SSA [32,57], were represented by relatively fewer studies in this review. In particular, fewer studies from francophone countries such as Benin, Congo, Niger, and Rwanda may be attributed to language barriers and the limited accessibility of non-English publications during the selection process. This discrepancy may reflect differences in research visibility, language accessibility, and publication coverage rather than actual research activity. Additionally, countries such as Benin and Congo report relatively low maize yields according to FAOSTAT in 2023 (https://www.fao.org/faostat/en/#data/QCL, accessed on 20 September 2025), which could indicate limited investment and institutional engagement in maize research and improvement programs. Overall, the distribution pattern highlights the uneven research intensity across SSA and the need for broader regional collaboration and inclusion to strengthen maize improvement initiatives.

The reduction in average maize yield across major production regions of sub-Saharan Africa (SSA) has been attributed to multiple factors, primarily abiotic and biotic stresses that are increasingly intensified by climate change [22,58,59,60]. The projected rise in the frequency and severity of climate-related stresses underscores the urgency for proactive and innovative research strategies to mitigate future impacts [61,62,63]. As a staple crop in SSA, maize remains highly vulnerable to these challenges due to its widespread cultivation and the heavy dependence of rural populations on it as a primary source of calories and household income [35,42,64]. Hence, developing resilient, high-yielding, and stress-tolerant maize varieties through innovative and collaborative breeding strategies is crucial to sustain maize productivity and food security in SSA under changing climatic conditions.

In SSA, land extensification, rainfed farming systems, and lower adoption of improved technologies are predominant among smallholder farmers [32,40,42,65]. This has led to substantial losses in maize production. Efforts to improve maize production in SSA have relied on strong collaboration among international research institutes, private partners, and National Agricultural Research Systems (NARS) [56,66,67]. The studies included in this review represent collaborative research efforts of many actors across SSA. These include maize breeders (experts), academic staff, and students across different levels of higher education, as well as private-sector enterprises working with support from non-governmental organizations (NGOs), including the Bill and Melinda Gate Foundation, the German Academic Exchange Service (DAAD), the Alliance for a Green Revolution in Africa (AGRA), USAID, etc.; government agencies and NARS; and international research institutes across SSA. Institutions such as CIMMYT, IITA, and WACCI served as major sources of parental materials, reflecting their advanced breeding programs, while National Agricultural Research Systems in different countries also contributed locally adapted parental lines for improvement. This broad participation underscores the critical role of coordinated engagement among international research centers, national institutions, private organizations, and academia in addressing food security challenges under increasing population pressure and climate variability in SSA.

### 4.3. Synthesis of Study Objectives Result

The predominance of studies focusing on yield improvement reflects the continued prioritization of yield as a central breeding objective in the region. Drought, heat stress, and pest and disease pressures have consistently constrained maize productivity across diverse agroecological zones [29,68,69]. Although substantial progress has been achieved through the development of stress-tolerant, pest- and disease-resistant, and high-yielding genotypes, the persistence of abiotic constraints and the emergence of new biotic threats highlight the need for sustained and adaptive breeding efforts [70,71,72]. Notably, many studies combined multiple objectives, addressing yield alongside other traits such as drought or pest resistance, demonstrating an integrated approach to tackling the complex and interrelated constraints affecting maize production [11,12,73]. Combining ability (CA) studies have played a crucial role in identifying parental lines with superior additive (GCA) and non-additive (SCA) gene effects, facilitating the development of hybrids with enhanced performance and resilience under stress conditions [74,75,76]. Such studies continue to inform breeding strategies by guiding the selection of promising parental materials for hybrid development and heterotic group classification.

Furthermore, research focused on nutritional improvement remains limited, despite the central role of maize in the diets of rural communities. The deficiency of essential amino acids and micronutrients in conventional varieties underscores the importance of biofortification as a complementary breeding goal [77,78,79]. Integrating nutrient-enhancing genes from donor parents through CA-based approaches, alongside improvements in nutrient uptake efficiency of cultivars and varieties, holds significant potential for enhancing the nutritional quality and food security contribution of maize in SSA [52,80,81]. Overall, the distribution of research focus observed in this review emphasizes that while yield improvement remains dominant, there is growing recognition of the need for multi-trait breeding strategies that simultaneously address productivity, resilience, and nutritional quality to ensure sustainable maize production under changing climatic conditions.

### 4.4. Synthesis of Parent Materials Result

The success of any breeding program and the effective introgression of desirable traits from a set of parental lines largely depend on the application of an appropriate mating design [1,2,3]. Each mating design has unique characteristics that determine the type, number, and genetic relatedness of the progenies obtained from the crosses [82,83]. Also, the choice of design is closely linked to the type and number of parental materials involved. From the selected studies, various mating designs were identified, each influencing the number and grouping of female and male parental lines used in hybrid development. In this study, inbred lines and open-pollinated varieties (OPVs) were both employed as parental materials. These materials differ in their genetic makeup and are used for distinct breeding objectives [4,15]. According to reports from the selected studies, parent materials are selected from pre-screened populations developed (inbred lines) or assembled (OPVs) for a specific trait and used in the transfer of the desired traits into hybrid progenies.

Inbred lines possess a narrow genetic base and are highly homozygous due to prolonged selfing over several generations [22,63,72]. Once near-complete homozygosity of alleles is achieved, the lines are selected and classified based on specific target traits for use in hybrid development [29,74]. In contrast, OPVs have a broad genetic base and are genetically heterogeneous, resulting from natural or uncontrolled cross-pollination [84,85,86]. Their inherent genetic variability confers wide adaptability across different environments, making them suitable for developing varieties with stable performance under diverse growing conditions. Thus, the genetic differences between inbred lines and OPVs play a crucial role in determining the combining ability, performance, adaptability and stability of the progenies in similar or contrasting environments.

The results revealed a predominance of GCA effects in crosses involving inbred lines, reflecting the combined additive contributions of both parents involved in the crosses. This outcome is expected, as heterosis in maize is optimally expressed when genetically distinct but homozygous parental lines are crossed to produce heterozygous hybrids [4,15]. Parents with greater genetic divergence are therefore more likely to generate hybrid vigor, underscoring the importance of combining ability analysis in identifying inbred lines with high genetic distance for hybrid development [19,87]. In crosses between inbred lines, allele segregation within full-sib progenies is relatively uniform due to the homozygous nature of the parents [88,89,90], in contrast to crosses involving inbred lines and OPVs or OPV × OPV combinations [15,85,86]. Consistent with this expectation, reviewed studies indicated that crosses involving OPV parental materials exhibited higher SCA effects than GCA effects [84,85,86], as seen in Table 3. Because OPVs are genetically heterogeneous and largely heterozygous, allele segregation in their progenies is non-uniform, resulting in greater variability in hybrid performance among progenies derived from similar parents across and within environments.

### 4.5. Synthesis of Mating Design Results

The grouping and number of participating lines differ across various mating designs. The predominance of the line × tester design in the reviewed studies can be attributed not only to its flexibility and efficiency in introgressing desired traits from well-characterized parents [69,91] but also to its feasibility in employing a large number of lines to be tested. Consequently, in this review across different studies in SSA, inbred lines were the most frequently used parental materials for the line x tester mating design. Due to their homozygosity, they provide more accurate estimates of GCA and SCA [90,92,93]. In some cases, as reported in this review, crosses between inbred lines and open-pollinated varieties (OPVs) were also conducted within the line × tester mating design [86,94]. This approach allows breeders to exploit the broad genetic base and heterozygosity of OPVs to enhance adaptability across diverse environments. It also improves the gene population and facilitates the accumulation of desirable traits across multiple genotypes.

In the case of the diallel mating design, the choice between full- and half-diallel crosses largely depends on the research objectives and the type of genetic information sought, as well as the number of parents to be involved in the hybridization scheme [1,82,95]. The full-diallel design, which includes reciprocal crosses, is preferred when cytoplasmic or maternal effects are of interest [71,96], while maternal effect is negligible in the half-diallel scheme. Based on findings from the reviewed studies employing diallel mating designs, crosses exhibiting high GCA across multiple locations were typically classified as potential single-cross hybrids or used as parents in three-way hybrid combinations [88,97,98]. The best-performing specific combinations were identified through their SCA effects and subsequently advanced to multi-environment trials for potential release [5,25,85,99]. In this mating design, the set of parental lines are evaluated for their combining ability through all possible crosses among themselves. However, a key limitation of the diallel design is that it requires extensive field space and resources for progeny evaluation as the number of parents increases, thereby restricting the number of parental lines that can be effectively tested in a single breeding program.

The North Carolina II (NCII), also known as the factorial design, is widely used to evaluate the combining ability of two distinct parental groups, typically classified as female and male sets [22,100,101]. In this design, each female line is crossed with all male lines, producing related progenies that share a common parent [21,75,77]. This facilitates the selection of widely adapted and stable parent lines suitable for use in various hybrid combinations. Owing to its efficiency and flexibility, the NCII design remains highly suitable for maize hybrid breeding programs, particularly for assessing parental divergence and stability across different environments to ensure consistent hybrid performance. Bhadmus et al. (2021) [102] and Osuman et al. (2022) [22] added that stable, single-cross hybrids identified across environments can be advanced to on-farm trials for potential release, while Udo et al. (2023) [103] recommended that they can be used as parents in three-way hybrid or synthetic combinations.

### 4.6. Synthesis of Combining Ability Estimates

Combining ability variance components were used to quantify the relative contributions of general combining ability (GCA) and specific combining ability (SCA) using Baker’s ratio [104,105]. This predictability ratio indicates the extent to which additive versus non-additive gene action governs trait expression and provides guidance for selecting appropriate hybrid breeding strategies [1]. The relative proportions of GCA and SCA, as reflected by Baker’s ratio (ranging from 0 to 1), also provide insight into the heritable potential of traits transmitted from parents to their offspring. High additive genetic variance, associated with GCA, corresponds to higher narrow-sense heritability [22], whereas a predominance of dominance variance, associated with SCA, is indicative of lower narrow-sense heritability [72]. The formulars below are used in estimating proportion of GCA and SCA, as well as narrow-sense heritability.

Baker’s ratio [104,105]

$$r=\frac{2\sigma^{2}gca}{2\sigma^{2}+\sigma^{2}sca}$$

where

*σ^2^ gca* = *gca* variance

*σ^2^ sca = sca* variance

Ranges:

0.7–1 (high additive gene action; GCA)

0.5 (equal GCA and SCA effects)

<0.5 (high dominance or epistatic effects; SCA)

Narrow-sense heritability (*h^2^*) [15,106]

$$h^{2}=\frac{Additive\;varince}{Phenotypic\;variance}$$

The heterosis and overall performance of progenies derived from controlled hybridization using specific mating designs are primarily determined through combining ability (CA) analysis [12,72]. This analysis provides essential insights for identifying and classifying parental materials into appropriate heterotic groups consistent with defined breeding objectives [68,91,107]. Findings from the reviewed studies revealed substantial variation in parental interactions, which significantly influenced hybrid performance across diverse environments and stress conditions. Hybrid progenies were evaluated under both optimal and stress environments to determine how parental lines interacted with environmental factors to influence progeny performance [13,75,108,109]. The magnitude of these interactions was quantified through the relative contributions of both parents, expressed as general combining ability (GCA) and specific combining ability (SCA). GCA reflects additive genetic effects, whereas SCA represents non-additive effects contributing to hybrid performance.

Across most studies, GCA effects were predominant, consistent with the frequent use of inbred lines as parental materials, reflecting their homozygosity and genetic uniformity [102,108,110]. Consequently, hybrid performance was largely influenced by the additive effects of both parents [60,88,111], while dominance and epistatic interactions occasionally limited the expression of certain traits in either of the parent lines. The inclusion criteria adopted in this review required clear characterization of parental materials, ensuring consistent and reliable interpretation of combining ability results across studies. The highest GCA effects were recorded for biotic stress tolerance and yield-related traits, underscoring the strong additive genetic control governing these characteristics. High GCA for yield traits indicates stable and heritable additive gene contributions [61,68,74,90]. This is an essential foundation for improving grain yield potential through hybridization. Similarly, for biotic stress tolerance, the observed trend reflects the additive effects of resistant and tolerant alleles present in the selected parental materials [6,13,112,113]. Notable SCA effects were also reported for yield and biotic stress tolerance, emphasizing the importance of selecting specific high-yielding or stress-tolerant donor parents to effectively exploit non-additive gene interactions for enhanced hybrid vigor [5,59,86]. Such hybrid progenies can contribute to the development of well-adapted and high-performing varieties suited to specific environments.

### 4.7. Heterogeneity and Sensitivity

The variation observed among the selected studies reflects the diversity of maize research across sub-Saharan Africa and the different challenges affecting production in the region. Differences among countries also indicate varying levels of participation and focus in maize improvement efforts. The strong emphasis on yield improvement, often prioritized over other objectives, helps explain yield reductions reported across environments due to multiple constraints. Rather than weakening this review, the observed heterogeneity highlights important trends and identifies key areas that require attention to improve maize productivity using combining ability approaches.

#### Sensitivity of the Results

To assess the robustness of the synthesized results, reporting trends were examined across studies that differed in mating designs, parental materials, numbers of trial evaluations, and geographic distribution. The alignment among study objectives, mating designs, and reported combining ability estimates provided a coherent framework for evaluating consistency in outcomes. Inbred lines were the predominant parental materials across regions and objectives and were largely associated with general combining ability (GCA) effects, whereas open-pollinated varieties (OPVs) were more commonly associated with specific combining ability (SCA) effects. Yield improvement was the most frequently targeted objective and was strongly influenced by GCA effects, while an increasing focus on nutritional improvement highlights the growing importance of maize biofortification. Overall, these patterns underscore the importance of combining ability analysis in the development of resilient maize cultivars for sub-Saharan Africa.

### 4.8. Reporting Bias

Despite improving methodological rigor, the included studies showed variability in the reporting of combining ability estimates, study objectives, and traits measured, as well as differences in the number of trial evaluations for each study. Application of the ROBINS-I framework highlighted recurring gaps in the consistency of reporting general and specific combining ability estimates and in linking parental performance to stated breeding objectives. These reporting differences cannot be standardized due to the above-stated variation but can be synthesized descriptively in Figure 9.

#### 4.8.1. Bias Report from Study Distribution

The number of studies varied markedly across the sub-Saharan Africa region, with Nigeria contributing the highest number (39 studies), while only 1 study each was reported from Benin, Congo, Niger, Rwanda, and Tanzania. Similarly, the intensity of trial evaluations differed across countries, with multiple locations reported in Nigeria, Ghana, and Zimbabwe, whereas Congo, Benin, and Zambia recorded relatively few trial evaluations. These patterns reflect regional differences in research capacity and levels of participation in maize improvement programs across the region.

#### 4.8.2. Bias Report from Study Objectives

The predominance of studies focused on yield improvement highlights its central importance to food security in sub-Saharan Africa. However, other reported objectives such as tolerance to abiotic stresses, resistance to biotic factors, and nutritional improvement address major constraints that contribute to yield reduction across the region [40,114]. Addressing these objectives alongside yield improvement is therefore essential for achieving sustained and effective gains in maize productivity.

#### 4.8.3. Bias Report from Mating Designs

The application of mating designs varied across the studies in both type and frequency. The line × tester design was most commonly used due to its flexibility, while half-diallel and North Carolina designs were applied at comparable levels; full-diallel was used in only a few studies. The choice of mating design largely depended on the breeder’s objectives, as well as the number and type of parental materials available. Full-diallel designs were typically adopted when maternal effects were of interest [71,96,115], whereas increasing the number of parents in both full- and half-diallel designs often resulted in large populations requiring extensive field evaluation, thereby limiting parental numbers. In contrast, line × tester and North Carolina designs were more accommodating of larger numbers of parental materials.

#### 4.8.4. Bias Report from Parent Material

Two main types of parental materials were used across the studies: inbred lines and open-pollinated varieties (OPVs), with inbred lines being used more frequently. This reflects the ease with which desirable traits, especially those controlled by additive genetic effects, can be transferred using inbred lines through appropriate mating designs [22,88,89]. However, OPVs, despite their greater variability due to segregation across generations, provide broad genetic diversity and are suitable for developing widely adaptable varieties [84,85,86], whereas inbred lines may be less stable across some environments [72,116].

#### 4.8.5. Bias Report from Combining Ability Estimate

The estimation of combining ability, expressed as the relative contribution of general combining ability (GCA) and specific combining ability (SCA), varied across studies depending on the traits measured and the trial evaluation conditions. Due to this heterogeneity, pooled effect measures were not applicable. Consequently, results were synthesized based on the authors’ conclusions regarding the predominant gene effects reported in each study.

## 5. Discussion

This review demonstrates substantial progress in maize improvement across SSA, driven by combining ability studies conducted under diverse ecological conditions and institutional collaborations. The distribution of research efforts across multiple countries and agroecological zones reflects the collective participation of national and international breeding programs in addressing regional production constraints. Reviewed studies pursued varied objectives, including yield enhancement, drought tolerance, pest and disease resistance, and improvement of nutritional quality. It is concluded that the predominance of yield improvement as an objective for many studies underscores the persistent challenges of abiotic and biotic stresses that continue to affect maize productivity in SSA. Given the central role of maize as a primary source of calories in many households across the region, there is a need to strengthen biofortification and nutritional improvement research using combining ability analyses to identify parental lines with favorable gene effects for key nutritional traits, enabling the development of nutritionally enhanced maize varieties without affecting agronomic performance.

It is also concluded that a large population of parental materials comprising inbred lines and open-pollinated varieties (OPVs) have been developed and utilized to identify parents with desirable genetic attributes. The use of well-structured mating designs has been an instrumental approach in assessing parental performance and estimating both general combining ability (GCA) and specific combining ability (SCA), which provide insight into additive and non-additive gene actions, respectively. Across studies, GCA effects were more associated with all studied traits and inbred line parent materials, indicating the predominance of additive gene action and the heritable nature of these traits. Meanwhile, significant SCA effects in some environments revealed the potential to exploit non-additive interactions for enhancing hybrid vigor and adaptability under variable environmental conditions.

Overall, the findings from this review highlight the importance of combining ability analysis as a guiding tool for parent selection, heterotic grouping, and hybrid development in maize breeding programs. The accumulated knowledge from these studies has contributed to the development of high-performing and resilient hybrids suitable for both optimal and stress-prone environments in SSA. Future breeding should apply QTL mapping after combining ability analysis to detect progenies carrying more desirable trait alleles in their genomes, thereby accelerating the selection and development of resilient maize hybrids.

## Figures and Tables

**Table 1:** Country of studies.

Country	Number of Studies	Percentage (%)
Benin	1	1
Congo	1	1
Ethiopia	15	16
Ghana	13	14
Kenya	7	8
Niger	1	1
Nigeria	39	42
Rwanda	1	1
South Africa	3	3
Tanzania	1	1
Uganda	3	3
Zambia	2	2
Zimbabwe	7	7
Grand Total	94	

**Table 2:** List of trial environments/evaluations.

Pest and Disease Stress	Water/Heat Stress	Fertilizer (N) Application	Location/Site
Artificial aflatoxin inoculation	Combined heat and drought stress	Delayed application (usually for Striga experiment)	Field
Artificial FAW infestation	Drought stress	Low application	Screenhouse
Artificial fusarium inoculation	Heat stress	Optimum application	
Artificial MLN inoculation	Optimum		
Artificial Striga infestation	Rainfed		
Artificial turcicum inoculation			
Natural (high) MSV pressure			
Natural (low) MSV pressure			
Natural FAW infestation			
Natural fusarium infection			
Natural Striga infestation			
Non-FAW infestation			
Non-MLN infestation			
Non-Striga infestation			
Non-turcicum inoculated			
Vector MSV Inoculation			

Abbreviations: FAW (fall armyworm), MLN (maize lethal necrosis), MSV (maize streak virus), N (nitrogen).

**Table 3:** Combining ability estimate in relation to parent materials.

Parent Materials/Crosses	Reported CA Estimates in Hybrid Evaluation
Inbred line/Inbred line (total)	549
GCA	452
GCA/SCA	4
SCA	93
Inbred line/OPV (total)	18
GCA	7
SCA	11
OPV/OPV (total)	13
GCA	6
SCA	7
Grand Total	580

Abbreviation: GCA (general combining ability), SCA (specific combining ability), OPV (open-pollinated variety).

## Data Availability

All data supporting this review are included in this article and its Supplementary files. Any additional information related to this study will be provided by the corresponding authors upon request.

## Supplementary Materials

The following supporting information can be downloaded at: https://www.mdpi.com/article/10.3390/genes17020168/s1.

## Abbreviations

The following abbreviations are used in this manuscript:

CA	Combining ability
CIMMYT	International Maize and Wheat Improvement Center
FAW	Fall armyworm
GCA	General combining ability
IITA	International Institute of Tropical Agriculture
MLN	Maize lethal necrosis
MSV	Maize streak virus
N	Nitrogen
NCII	North Carolina II
OPVs	Open-pollinated varieties
PICOTS	Population, Intervention, Comparator, Outcomes, Time, and Settings
QTL	Quantitative trait loci
RCBD	Randomized complete block design
SCA	Specific combining ability
SSA	Sub-Saharan Africa
WACCI	West Africa Centre for Crop Improvement
