# Supplementary material for: Combining Ability in Maize Breeding Programs in Sub-Saharan Africa: A Systematic Review
Source: Genes (Basel). 2026 Jan 30;17(2):168. doi: 10.3390/genes17020168 (PMC12940587; doi:10.3390/genes17020168)
Supplement: Supplementary file 1 [file genes-17-00168-s001.zip › RoBINS_I_Traffic_Light_A4.pdf]

Risk of bias domains (Page 1 of 3)

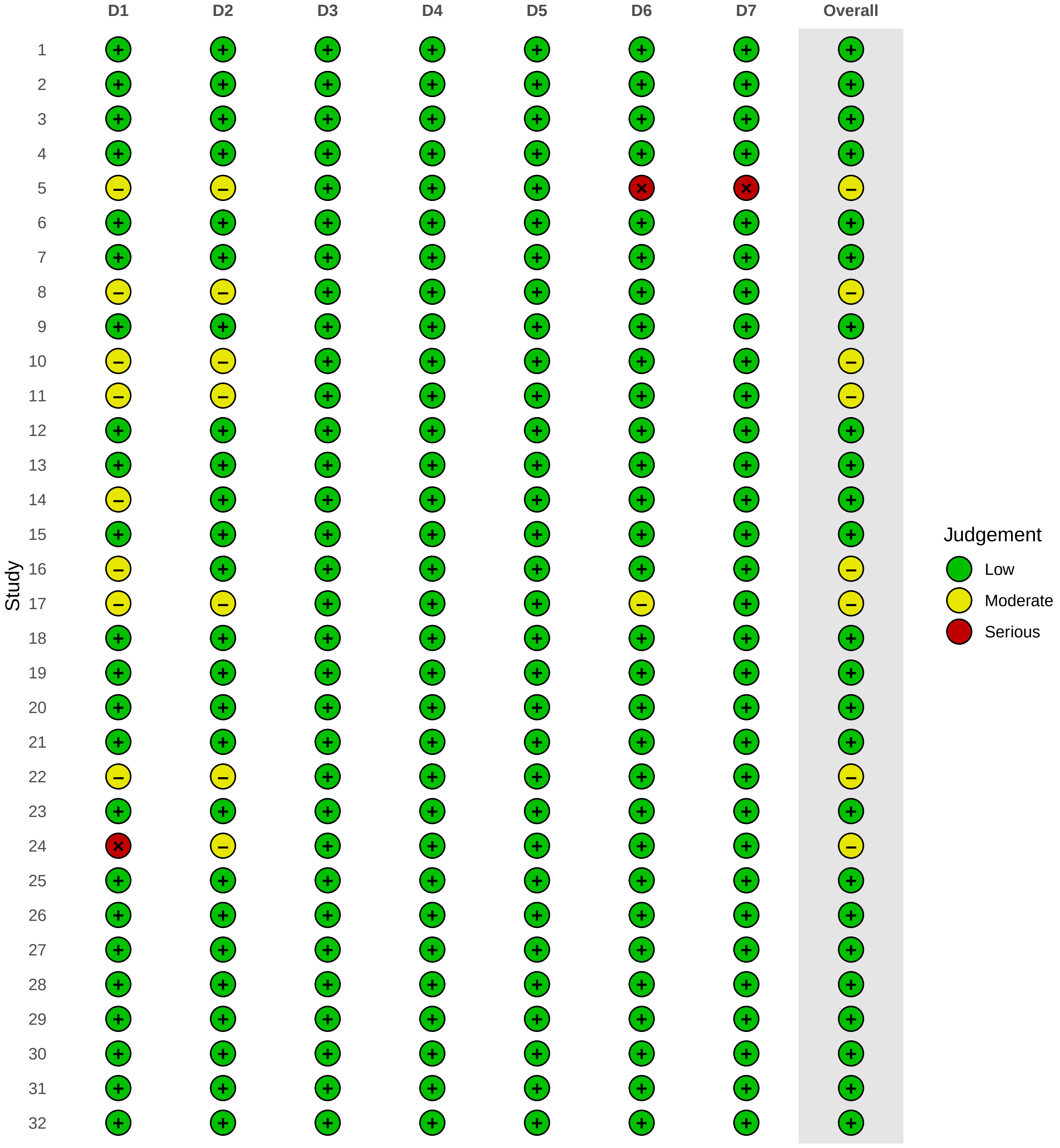

Domains:
D1: Bias due to confounding.
D2: Bias due to selection of participants.
D3: Bias in classification of interventions.
D4: Bias due to deviations from intended interventions.
D5: Bias due to missing data.
D6: Bias in measurement of outcomes.
D7: Bias in selection of the reported result.

Risk of bias domains (Page 2 of 3)

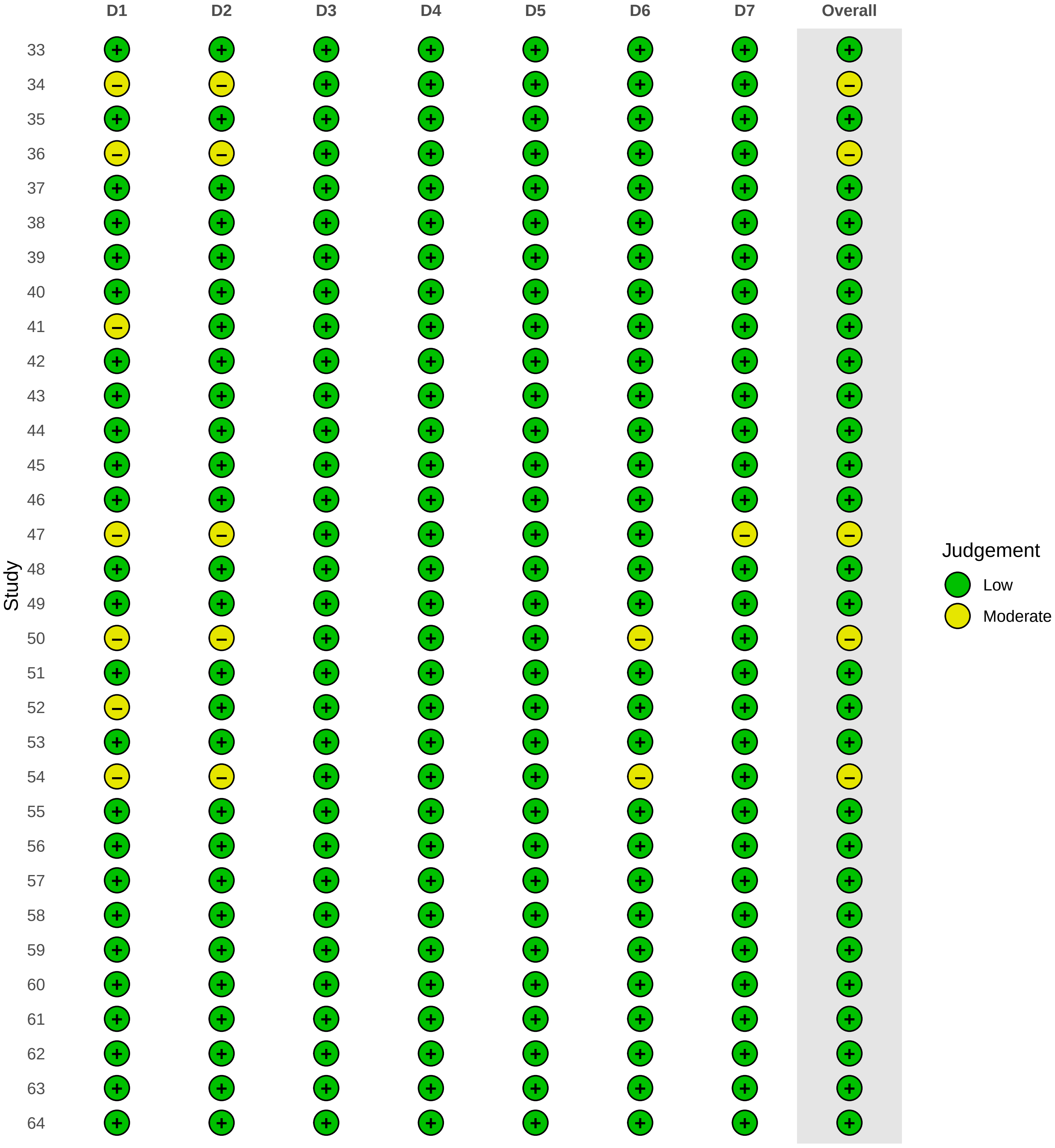

Domains:
D1: Bias due to confounding.
D2: Bias due to selection of participants.
D3: Bias in classification of interventions.
D4: Bias due to deviations from intended interventions.
D5: Bias due to missing data.
D6: Bias in measurement of outcomes.
D7: Bias in selection of the reported result.

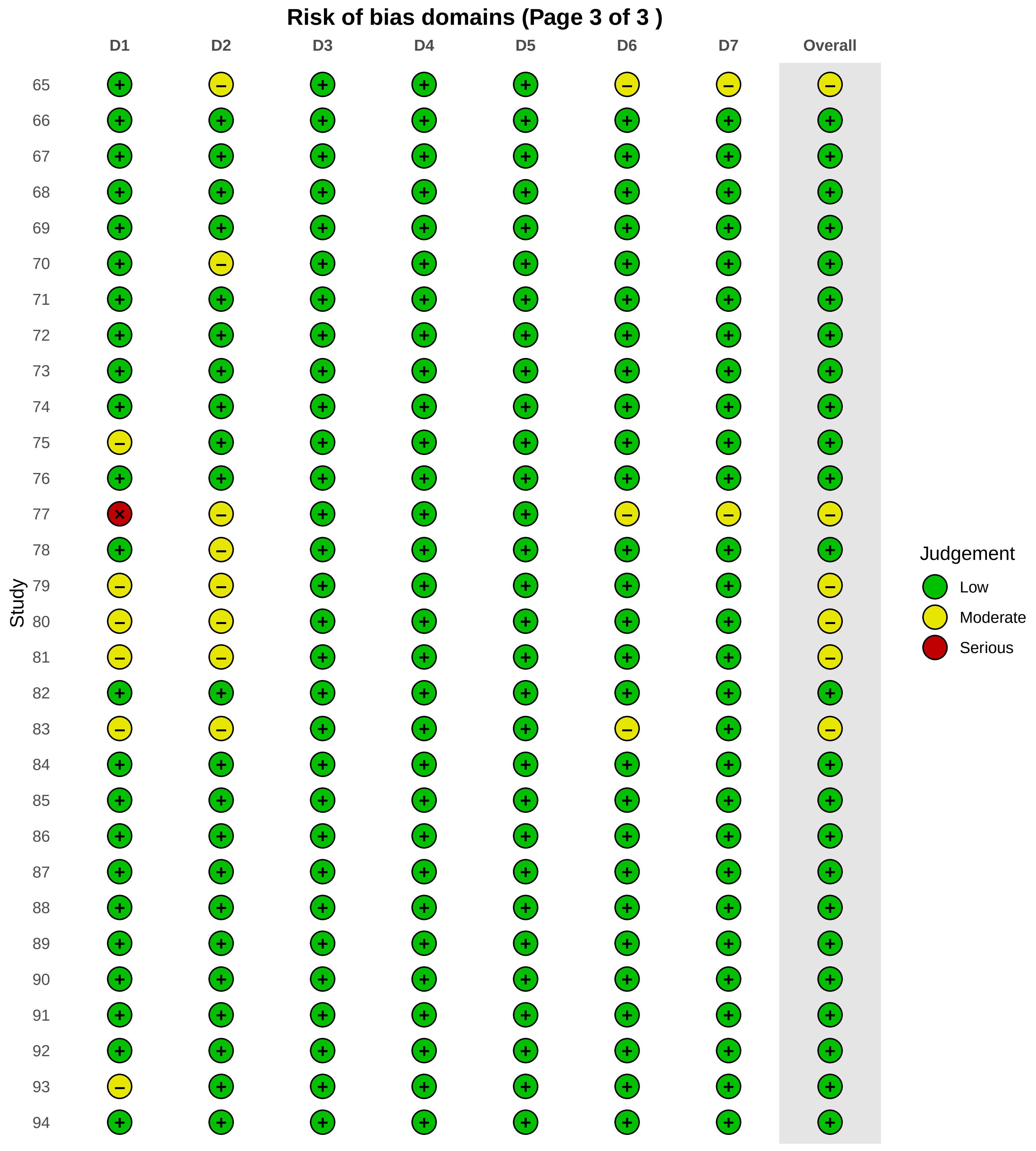

Domains:
D1: Bias due to confounding.
D2: Bias due to selection of participants.
D3: Bias in classification of interventions.
D4: Bias due to deviations from intended interventions.
D5: Bias due to missing data.
D6: Bias in measurement of outcomes.
D7: Bias in selection of the reported result.
